# Healthcare professionals’ views on feedback of a patient safety culture assessment

**DOI:** 10.1186/s12913-016-1404-8

**Published:** 2016-06-17

**Authors:** Nicolien C. Zwijnenberg, Michelle Hendriks, Janneke Hoogervorst-Schilp, Cordula Wagner

**Affiliations:** Netherlands Institute for Health Services Research, P.O. Box 1568, 3500 BN Utrecht, The Netherlands; Department of Public and Occupation Health, EMGO + Institute for Health and Care Research, VU University Medical Center (VUmc), Van der Boechorststraat 7, 1081 BT Amsterdam, The Netherlands

**Keywords:** Healthcare professionals’ views, Feedback, Performance assessment, Patient safety culture

## Abstract

**Background:**

By assessing patient safety culture, healthcare providers can identify areas for improvement in patient safety culture. To achieve this, these assessment outcomes have to be relevant and presented clearly. The aim of our study was to explore healthcare professionals’ views on the feedback of a patient safety culture assessment.

**Methods:**

Twenty four hospitals participated in a patient safety culture assessment in 2012. Hospital departments received feedback in a report and on a website. In a survey, we evaluated healthcare professionals’ views on this feedback and the effect of additional information about patient safety culture improvement strategies on the appraisal of the feedback. 20 hospitals participated in part I (evaluation of the report), 13 hospitals participated in part II (evaluation of the website).

**Results:**

Healthcare professionals (e.g. members of staff and department heads/managers) rated the feedback in the report and on the website positively (average mean on different aspects = 7.2 on a scale from 1 to 10). Interpreting results was sometimes difficult, and information was sometimes lacking, like specific recommendations and improvement strategies. The provision of additional general information on patient safety culture improvement strategies resulted only in a higher appraisal of the attractiveness (lay-out) of the report and the understandability of the feedback report. The majority (84 %) of the healthcare professionals agreed or partly agreed that the feedback on patient safety culture stimulated actions to improve patient safety culture. However, a quarter also stated that although the feedback report provided insight into the patient safety culture, they did not know how to improve patient safety culture in their hospital.

**Conclusions:**

Healthcare professionals seem to be positive about the feedback on patient safety culture and its effect on stimulating patient safety culture improvement. To optimally tune feedback on patient safety culture towards healthcare professionals, the following might help: 1) pay attention to the understandability of outcomes for its intended users; and 2) create feedback that is tailored towards specific hospital departments.

## Background

Patient safety is an important indicator of quality of healthcare [1]. Therefore, enhancing and guaranteeing patient safety is a high priority for healthcare providers. Today, promoting a positive patient safety culture has become one of the pillars for improving patient safety [2]. Patient safety culture can be described as: the product of individual- and group values, attitudes, perceptions, competencies, and patterns of behaviour that determine the commitment to, and the style and proficiency of, an organisation’s health and safety management [2, 3]. Enhancing an open culture in which errors and adverse events can be discussed may help reduce medical errors, and thereby improve patient safety [3].

To assess patient safety culture, several measurement tools have been developed in the past decade and are widely used [4–6]. For example, the Hospital Survey on Patient Safety Culture (HSOPSC) [2] or the Safety Attitude Questionnaire (SAQ) [6]. Healthcare providers are supported to identify areas for improvement by using these measurement tools, which may stimulate change in patient safety culture. A variety of strategies can be used by healthcare providers to make improvements in patient safety culture. For example, leadership walk rounds - an informal method for senior leaders to talk with front-line staff about safety issues and show their support for safety - or training programmes focusing on teamwork and collaboration [7].

An important precondition to improve patient safety culture is that outcomes of patient safety culture assessments are relevant and are presented clearly for the intended users, i.e. healthcare providers. Until now, several studies have been performed on how quality reports - containing information on quality of care of healthcare providers - can be best presented to consumers [8–20]. Only a few studies have focused on how quality reports can be best presented to healthcare professionals [21–25]. For example, a study of Allwood, Hildon & Black (2013) [23] explored clinicians’ comprehension of and preferences for format and content in displaying provider outcomes. It showed that healthcare professionals prefer formats they are familiar with (such as bar charts or numeric tables) and want to have data in more than one format. Since evidence is still scarce in this field, and quality measurement comprises many dimensions (e.g. measuring patient experiences, mortality rates, patient safety culture), more insight into how healthcare professionals appraise outcomes of quality performance assessments is desirable.

This study explores healthcare professionals’ views on feedback of a patient safety culture assessment. Patient safety culture was assessed within 24 hospitals in the Netherlands at the end of 2012, with feedback being provided to them. As change in patient safety culture can be seen as an intended objective of this performance assessment [1], we explored healthcare professionals’ opinions about the effect of feedback on improving patient safety culture. We also explored whether additional information on general patient safety culture improvement strategies resulted in a higher appraisal of the feedback information.

Two research questions will be addressed in this evaluation study:

1. What are healthcare professionals’ views on feedback of patient safety culture?
2. Will providing additional general examples of patient safety culture improvement strategies result in a higher appraisal of the feedback information?

## Methods

**Fig. 1:**
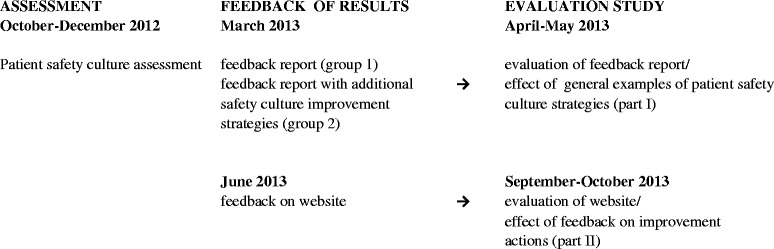
Overview of data collection during patient safety culture assessment and evaluation study

An overview of the data collection during the patient safety culture assessment and current evaluation study is provided in Fig. 1.

### Patient safety culture assessment

At the end of 2012, 24 hospitals participated in a patient safety culture assessment. The validated Dutch version of the AHRQ’s Hospital Survey on Patient Safety Culture (HSOPSC), COMPaZ, was used to measure patient safety culture [26]. The COMPaZ contains 40 items on patient safety culture with a five-point Likert scale (1 = disagree strongly/never to 5 = agree totally/always). These items represent 11 dimensions of safety culture (see Table 1). Furthermore, the questionnaire contains seven items concerning respondents’ background variables (e.g. respondent’s department in a hospital) and two outcome measures: a patient safety grade (on a five-point Likert scale; 1 = excellent to 5 = worse), and the number of errors reported by the respondent in the previous twelve months. In total, 390 hospital departments of 24 hospitals participated in the patient safety culture assessment and 6,605 questionnaires were returned (a response rate of 60 %). The response rate per hospital varied between 32 and 85 %. The average response rate on department level was 60 % [27].

**Table 1:** Information about the patient safety culture assessment and feedback that was provided

11 dimensions of patient safety culture are measured with the COMPaZ: • teamwork across departments • teamwork within departments • correct hospital handoffs and transitions • frequency of event reporting • non-punitive response to error • communication openness • feedback on and learning from errors • supervisor/manager expectations about safety • management support • sufficient staffing • general perceptions of patient safety
The feedback report contained: • general information about the patient safety culture assessment • general information and the stages of development of patient safety culture • results on department and hospital level - table with absolute figures of respondents’ function - bar chart with reported number of errors - bar chart with patient safety grade - table with mean scores on the 11 patient safety culture dimensions - numeric tables with percentage of positive responses (score 4 and 5) for every item • general summary and recommendations • (optional) appendix with general examples of improvement strategies for patient safety culture (e.g. performing walk rounds, establishing a quality commission)
The website contained: • a homepage with general information about the patient safety culture assessment and links to - general information and the stages of development of patient safety culture - general examples of improvement strategies for patient safety culture - a digital download of the feedback report (see above) • a menu (toolbar) with results on department level and benchmark options - table with absolute figures of respondents’ function (no benchmark options) - bar chart and table with reported number of errors (benchmark options) - bar chart and table with patient safety grade (benchmark options) - table with mean scores on the 11 patient safety culture dimensions (benchmark options) - bar chart and table with percentage of positive responses (score 4 and 5) for every item (benchmark options) • benchmark options for hospital departments on the website - results of their own hospital (hospital level) - results of the same type of specialism in other hospitals (14 different groups were developed) - results of different types of hospitals (general, teaching or categorical hospital) - results of all respondents of participating hospitals

### Feedback of results

Feedback to the hospital departments was provided in a report and on a website. In March 2013, departments within the 24 participating hospitals that had a sufficient response rate (≥60 %) received a feedback report containing their own results and the results of their hospital (see Table 1 for the content of this feedback report). To evaluate the effect of additional information on patient safety culture improvement strategies, two versions of the feedback report were created. Departments within 12 hospitals received a feedback report with an appendix containing descriptions of general examples of improvement strategies for patient safety culture (e.g. performing walk rounds, establishing a quality commission). These departments were selected randomly. Randomization on the hospital level ensured that departments in the same hospital received the same content of the feedback report. This was important to prevent contamination between departments and type of feedback.

**Fig. 2:**
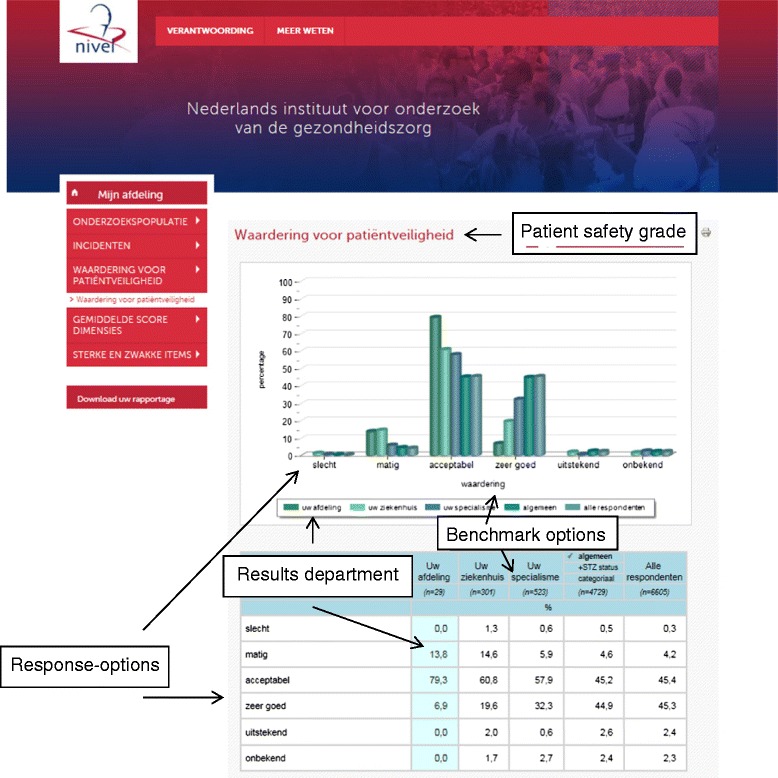
Feedback on website: results of the patient safety grade

In June 2013, a website was launched providing hospital departments with their own results and the opportunity to benchmark their results with others (see Table 1 for information about the website, Fig. 2 provides a screenshot of the website). The additional information about patient safety culture improvement strategies was available for all hospital departments on the website.

### Evaluation study

The current evaluation study consisted of two parts: an evaluation of the report in which the effect of additional information of patient safety culture improvement strategies was also tested, and an evaluation of the website. Initially, we aimed to receive input from department members who received feedback. However, a pilot study on the evaluation of the report in one hospital resulted in a relatively low response rate on the department level (36 %). Consequently, we focused primarily on input from the contact persons in all hospitals who coordinated the patient safety culture assessment in their hospital. These were often staff members or advisors in the field of quality improvement.

#### Part 1: evaluation of the feedback report

A short questionnaire was developed for the evaluation of the report. This questionnaire contained four items about the content of the report (e.g. rating the report on relevance and understandability, see Table 2), and two items about preferences related to web-based information for additional benchmark opportunities (these data are not reported here).

**Table 2:** Evaluation of feedback in the report and on the website

		Report (*n* = 20)	Website (*n* = 13)
Item		Mean (CI) Scale 1–10	Mean (CI) Scale 1–10
1	I think that the patient safety culture feedback report/website is…		
	Understandable	6.8 (5.9–7.6)	7.3 (6.2–8.3)
	Interesting	7.6 (7.1–8.1)	7.5 (6.9–8.2)
	Useful	7.0 (6.2–7.8)	7.2 (6.3–8.1)
	Relevant	7.6 (7.2–8.0)	7.2 (6.2–8.1)
	Well-ordered	7.1 (6.5–7.7)	6.8 (5.9–7.8)
	Complete	7.2 (6.6–7.7)	7.1 (6.3–7.9)
	Attractive (lay-out)	7.3 (6.8–7.7)	7.3 (6.6–7.9)
	Total	7.2 (6.8–7.6)	7.2 (6.4–7.9)
	**Report**	*N* (%)	
2	Do you miss any information in this report?		
	Yes	12 (60 %)	
3	Which statement reflects the patient safety culture report most:		
	we now see suggestions to improve patient safety culture	11 (55 %)	
	we now have insight, but we do not know how to improve	5 (25 %)	
	this report has brought us little new information	1 (5 %)	
	different statements^a^	3 (15 %)	
4	Do you recognise your department/hospital in the results of this report?		
	no	0 (0 %)	
	partially	9 (47 %)	
	yes, absolutely	10 (53 %)	
	**Website**		*N* (%)
2	Do you miss any information on this website?		
	Yes		3 (27 %)
3	General comments on the website [open-ended question]		-
4	Why has your hospital performed a patient safety culture assessment (more options allowed)		
	diagnose safety culture		13 (100 %)
	evaluate patient safety interventions & track changes over time		7 (54 %)
	conduct internal/external benchmark		5 (38 %)
	fulfil directives or regulatory requirements		1 (8 %)
5	Have departments taken action to improve the patient safety culture?		
	no		1 (8 %)
	action will be taken		5 (42 %)
	yes		6 (50 %)
6	Has the feedback on patient safety culture stimulated this action?		
	no, not at all		2 (17 %)
	partly		5 (42 %)
	yes, absolutely		5 (42 %)
7	How can this feedback contribute (more) to action being taken? [open-ended question]		-

^a^Aggregated scores on the hospital level were calculated when more questionnaires of one hospital were returned (e.g. from different department members). The following directives were followed for aggregating the results. Evaluation of the feedback report: item 1; average scores were calculated for every aspect; item 2; answer ‘yes’ was used when one respondent marked this option; item 3; the extra option ‘different statements’ was constructed when different options were marked and item 4; answer ‘partly’ was used when different options were marked. For the evaluation of the website, aggregation of the results was not necessary as only the contact persons returned the questionnaire

The questionnaire was sent by email to the contact persons of the 24 hospitals that participated in the patient safety culture assessment. They were invited for a telephone interview performed by one of the authors (NZ) to discuss the questionnaire. Various response patterns spontaneously occurred: Some contact persons participated in the telephone interview themselves, and sometimes also disseminated the questionnaire to department members at our request. Some contact persons completed one single questionnaire with input from department members who received a feedback report. A few contact persons preferred not to participate in the telephone interview themselves, but passed on the invitation to complete this questionnaire to one or more department members. Department members, mainly department heads/managers, were able to return the questionnaire by email or post.

#### Part 2: evaluation of the website

Three months after the launch of the website, all contact persons were again invited to return a short questionnaire by post/email or, if preferred, to participate in a telephone interview conducted by one of the authors (NZ) to discuss the questionnaire. The questionnaire contained three items about the content of the website (e.g. rating the website on relevance and understandability) and four items on actions that were taken (or not taken) to improve the patient safety culture and the role of the feedback information in this (see Table 2). This time, only the contact persons returned the questionnaire.

Ethical approval of the evaluation study was not necessary as research by means of surveys that are not taxing and/or hazardous for participants (i.e. the once-only completion of a questionnaire containing questions that do not constitute a serious encroachment on the person completing it) is not subject to the Dutch Medical Research Involving Human Subjects Act (WMO). No written informed consent was therefore obtained from the participants in our study. Subjects were free to respond to the questionnaire and they were informed about the aim of the survey.

### Analyses

As a result of the different response patterns, analyses were performed on the hospital level. When more questionnaires were returned from one hospital (e.g. questionnaires from different department members), we calculated aggregated scores on the hospital level (see footnote Table 2 for directions that were followed).

Descriptive analyses were performed using STATA version 12.1. Qualitative data (respondents’ explanations on given answers, general comments and open-ended questions) were screened and, where possible, clustered into themes by one researcher (NZ). For example, on the item ‘Do you miss information in the feedback report?’ answers like ‘more information on interpretation of benchmark results’ and ‘more simplistic explanations’ were categorized into the theme ‘more information about interpretation of results or context’. To evaluate the effect of the provision of additional information on patient safety culture improvement strategies, answers of hospitals with this additional information vs. hospitals without this additional information were compared with the Wilcoxon Mann Whitney test or the Fisher’s exact test.

## Results

### Evaluation of the feedback report

In total, we received input from 20 of the 24 hospitals on the feedback report (evaluation study part 1). Eight questionnaires contained input from the contact person only (40 %), eight questionnaires contained input from both the contact person and department members (40 %) and four questionnaires contained input from different department members (20 %). Table 2 shows healthcare professionals’ views on the feedback report. Mean scores on the seven aspects on which the report was rated ranged from 6.8 (understandable) to 7.6 (interesting and relevant). Some respondents explained that the interpretation of results of negatively formulated items, for example “Hospital departments do not coordinate”, was difficult. The majority of the respondents (60 %) indicated that certain information in the report was lacking (item 2). Aspects that were mentioned concerned: a) specific conclusions/recommendations or specific patient safety culture improvements for their department/hospital; b) a list of the top ten weak and strong points for their department/hospital; c) the presentation of the number of participants who agreed/disagreed with items (in the report only the percentage of positive responses on an item was displayed); d) more benchmark opportunities; or e) more information about the interpretation of results or context. Some respondents mentioned also that a report with only the results on the hospital level was lacking (this report was available afterwards on the website).

All respondents, partly (47 %) or completely (53 %) agreed that the results in the feedback report reflected actual practice (item 4). Some respondents explained that the results were as expected and reflected their current patient safety culture or that the results confirmed results of other quality assessments tools that they had used. Others stated that they were pleasantly surprised by the results.

A small majority of the respondents (55 %) indicated that the feedback report helped them see how to improve patient safety culture (item 3). A quarter of the respondents (25 %) selected the statement “we now have insight into patient safety culture, but we do not know how to improve patient safety culture”.

### The effect of additional examples of patient safety culture improvement strategies

Eleven of the 20 responding hospitals (55 %) received a feedback report containing an appendix with general examples of patient safety culture improvement strategies. Generally, the hospitals who received this additional information were slightly more positive on several aspects of the feedback report than hospitals who did not receive this additional information (see Table 3). The groups differed significantly on two aspects. Hospitals that received additional information were more positive about the understandability (M = 7.3) and the attractiveness (M = 7.6) of the feedback report than hospitals who did not receive this additional information (M = 6.1 and M = 6.8, respectively). In both groups, some respondents indicated that specific conclusions/recommendations or specific patient safety culture improvements were lacking.

**Table 3:** The effect of additional general information on patient safety culture improvement strategies

Item		Group 1: feedback report (*n* = 9)	Group 2: feedback report with appendix (*n* = 11)	*P*-value
		Mean (CI)	Mean (CI)	
		Scale 1–10	Scale 1–10	
1^a^	I think that the patient safety culture feedback report is….			
	Understandable	6.1 (4.8–7.5)	7.3 (6.0–8.6)	0.04*
	Interesting	7.6 (7.0–8.1)	7.6 (6.7–8.6)	0.53
	Useful	6.8 (3.0–7.5)	7.2 (5.8–8.6)	0.10
	Relevant	7.3 (6.8–7.9)	7.8 (7.3–8.3)	0.20
	Well-ordered	6.7 (5.9–7.4)	7.5 (6.4–8.5)	0.12
	Complete	6.9 (6.2–7.6)	7.4 (6.6–8.2)	0.45
	Attractive (lay-out)	6.8 (6.3–7.3)	7.6 (7.0–8.3)	0.04*
		*N* (%)	*N* (%)	
2^b^	Do you miss any results in this report?	5 (56 %)	7 (64 %)	
	Yes			1.000
3^b^	Which statement reflects the patient safety culture report best:	6 (67 %)	5 (46 %)	0.49
	we now see suggestions to improve patient safety culture	1 (11 %)	4 (36 %)	
	we now have insight, but we do not know how to improve	2 (22 %)	1 (9 %)	
	this report has brought us little new information different statements	-	1 (9 %)	

^a^results of the Wilcoxon Mann Whitney test

^b^results of the Fisher’s exact test

*P<0.05

### Evaluation of website

We received input from 13 of the 24 hospitals on the evaluation of the website (evaluation study part 2; see Table 2). Mean scores on seven aspects on which the website was rated ranged from 6.8 (well-ordered) to 7.5 (interesting) (see item 1, Table 2). About a quarter (27 %) of the respondents indicated that they missed certain information on the website (item 2). Concerning information that was lacking, one respondent missed the option to compare departments of their hospital, another respondent mentioned that it was not always clear on which underlying items scores in graphics or tables were based (these items were not directly visible).

### The effect of feedback on patient safety culture improvement

All hospitals performed the patient safety culture assessment to assess the current patient safety culture (item 4, see Table 2). About half of the respondents (54 %) said they also performed the assessment to evaluate interventions and track changes over time.

About half of the respondents (50 %) indicated that they have taken action to improve patient safety culture (six months after receiving the feedback report and three months after the launch of the website, item 5). One respondent explained, for example, that periodic meetings between hospital departments were being organised. Another respondent mentioned that results of the assessment had been discussed within the teams, which could also be seen as an intervention as such. More attention for reporting errors with corresponding analysis and improvements was mentioned by another respondent. Another 42 % of the respondents indicated that they intended to take actions to improve patient safety culture. Some of these respondents explained that they were in the middle of the process of discussing the outcomes and one respondent expressed: “*To get teams to take action and self*-*improve is very difficult*”.

The great majority (84 %) of the respondents indicated that the feedback on patient safety culture had partly or wholly stimulated actions to improve patient safety culture, 17 % disagreed with this (item 6). As one of the respondents in favour of this statement explained: “*The patient safety culture assessment resulted in bottlenecks becoming clear*”. One opponent explained that the number of professionals that had participated in the patient safety culture assessment was low, and some others expressed that the feedback did not provide new information compared to other quality assessment tools they were using.

With regard to the question concerning whether the current feedback could contribute (more) on taking action, some respondents expressed that it is mainly an internal business whether action will take place, as clearly expressed by the following respondent: “*I think that the feedback cannot by any means contribute more than is the case now*. […] *The most important is that the hospital itself stimulates that these results are open for discussion and work out which actions are needed to create an open culture*”.

## Discussion

### Main findings

This study explored healthcare professionals’ views on feedback of a patient safety culture assessment. Hospitals that participated in this assessment received feedback in a report and on a website. In a survey, we evaluated staff- and department members’ (e.g. quality advisors and department heads/managers) views on this feedback. Additionally, we studied the effect of additional information on general patient safety culture improvement strategies on the appraisal of the feedback.

Generally, healthcare professionals rated the feedback on patient safety culture in the report and on the website positively on aspects such as relevant and interesting information. An important finding, however, was the relatively low score on the understandability of the feedback report compared to other aspects and compared to the score on this aspect for the website. Some respondents explained that they had difficulty interpreting the results of negatively formulated items (e.g. ‘hospital departments do not coordinate’). In the feedback report, the percentage of positive responses was presented for every item. For negatively formulated items, this means that the percentage of respondents who disagreed with the statement was presented. This might cause confusion and problems in interpreting results correctly and probably explains the lower score on understandability of the feedback report. For the website, negatively formulated items were reformulated positively (e.g. ‘hospital departments do not coordinate’ becomes ‘hospital departments coordinate’).

It is known that the understandability and usability of performance results is influenced by the presentation approach of information [23, 24, 28]. In the feedback report and on the website, bar charts and numeric tables were used as presentation formats. These could be characterised as familiar presentation formats for healthcare professionals [23]. It is of interest to further explore which presentation formats will benefit healthcare professionals most in effectively using feedback on patient safety culture or other quality dimensions.

Including additional general examples of patient safety culture improvement strategies did not result in an obvious higher appraisal of the feedback information. Only the attractiveness (lay-out) of the report and understandability of the information was rated higher. The fact that the additional appendix included several richly-coloured pictures might explain the higher appraisal of the attractiveness of the report.

An interesting finding was that specific conclusions/recommendations and specific improvement strategies for their department/hospital were found lacking, both in the group with additional general information on patient safety culture improvement strategies and the group without. It is known that by providing customised or tailored information, the meaning of information can be highlighted [8]. In the field of behavioural change studies, Noar and colleagues (2007) [29] showed that individually tailored health materials are more effective than generic materials in promoting behavioural change (e.g. physical exercise). In this perspective, tailoring outcomes of patient safety culture assessments and patient safety culture improvement strategies on the department level might likewise result in information that is more meaningful for the intended users.

The majority of respondents were of the opinion that the feedback partly or wholly contributed to improvements in patient safety culture. However, a quarter of the respondents also stated that although the feedback report provided insight into the patient safety culture, they do not know how to improve patient safety culture in their hospital. To actually achieve improvements in patient safety culture, hospitals themselves have a vital role to play in transforming survey results into actions [1], as was also observed by some respondents. For hospitals, an important aspect to keep in mind is that the patient safety culture assessment is just the beginning of realising change in this area, rather than the final destination. In addition to making meaningful outcome information available, investments in human capital are essential for creating an infrastructure for continuous improvements [1]. A useful strategy to be used by hospitals to identify effective improvement strategies is to set up a focus group with a representative sample of respondents who participated in a patient safety culture assessment [30]. A systematic review of Morello et al. (2013) [7], examining the impact of several patient safety culture improvement strategies, showed that strong evidence supporting best practices in patient safety culture improvement is still lacking. This might be linked to patient safety culture being a complex phenomenon [31]. To gain more insight into effective improvement strategies, therefore, it is important to examine which patient safety culture improvement strategies are used by different hospitals, and evaluate their effectiveness. Again, tailoring the feedback outcomes and patient safety culture improvement strategies might also result in a more effective use of the feedback in achieving patient safety culture improvement.

### Limitations of the study

This study explores the views of healthcare professionals - the intended users - on feedback of a patient safety culture assessment. Some limitations of the study exist, however. The first limitation is the small sample size (20 hospitals) of this study. We have chosen to focus primarily on the contact persons that had a coordinating role during the safety culture assessment. These were often staff members or advisors in the field of quality who were frequently involved in communicating the feedback reports to the hospital departments or the Board of Directors. As a result of the different response patterns that spontaneously emerged in part 1 of our evaluation study (individual responses of contact persons, a joint response of contact persons and department members, or individual responses of department members), analysing results on the hospital level was necessary. As a result of the small sample size and as participants were not randomly chosen, representativeness might be harmed and positive sampling bias might have occurred. Our participants may be more interested in topics on quality improvement than the average healthcare professional and might therefore also react more positively to feedback reports, or even be more critical.

The effects of feedback on (planned) actions to improve patient safety culture, which was part of the study in part 2 of our evaluation, have to be interpreted with some caution. Our results are based on participants’ perceptions rather than on the actual measurement of improvement actions undertaken. Finally, the questionnaires used to evaluate the patient safety culture report and website were quite limited. The use of short questionnaires, however, was a deliberate choice to reduce the workload to fulfil the surveys. Taking all these limitations into account, caution is warranted by the interpretation and generalisation of our study results. Our results may therefore be best characterised as a first impression of professionals’ views on feedback on patient safety culture.

## Conclusions

At first sight, healthcare professionals seem to be positive about feedback on patient safety culture and its effect on stimulating patient safety culture improvement. To optimally tune feedback on patient safety culture to healthcare professionals to stimulate change, the following might help: 1) pay attention to the understandability of outcomes for its intended users; and 2) create feedback that is tailored towards specific hospital departments. For hospitals, an important aspect to keep in mind is that the patient safety culture assessment and feedback on the outcomes are just the beginning of realising change in this area, rather than the final destination.

### Availability of data materials

The datasets supporting the conclusions of this article are available in the DANS (Data Archiving and Networked Services) repository http://dx.doi.org/10.17026/dans-2ae-h7kc.
